# Gender discrepancy in the predictive effect of metabolic syndrome and its components on newly onset cardiovascular disease in elderly from rural China

**DOI:** 10.1186/s12877-021-02393-6

**Published:** 2021-09-25

**Authors:** Shasha Yu, Xiaofan Guo, GuangXiao Li, Hongmei Yang, Liqiang Zheng, Yingxian Sun

**Affiliations:** 1 Department of Cardiology, First Hospital of China Medical University, 155 Nanjing North Street, Heping District, 110001 Shenyang, China; 2 Department of Clinical Epidemiology, Institute of Cardiovascular Diseases, First Hospital of China Medical University, 110001 Shenyang, China; 3 Department of Clinical Epidemiology, Shengjing Hospital of China Medical University, 110004 Shenyang, China

## Abstract

**Background:**

This study aimed to estimate whether metabolic syndrome (MetS) and its components could be used to predict cardiovascular disease (CVD) in a longitudinal analysis in a rural elderly Chinese population.

**Method:**

At baseline during 2012–2013, a total of 2486 elderly from rural Chinese were enrolled and were followed up during 2015–2017. Stroke and coronary heart disease (CHD) were included in CVD and were diagnosed by clinicians. The National Cholesterol Education Program Adult Treatment Panel III (NCEP ATP III), the American Heart Association/National Heart, Lung, and Blood Institute (AHA/NHLBI) and the International Diabetes Federation (IDF) criteria were used to define MetS separately.

**Result:**

Hazard ratios adjusting for CHD, stroke and CVD in those with MetS using the NCEP ATP III criteria in females were 1.27 (95 % CI 0.73, 2.21), 1.54 (95 % CI 0.99, 2.40) and 1.45 (95 % CI 1.00, 2.10), respectively; 1.33 (95 % CI 0.77, 2.32), 1.44 (95 % CI 0.92, 2.25) and 1.36 (95 % CI 0.94, 1.97), respectively, with the AHA/NHLBI criteria; and 1.10 (95 % CI 0.89,1.36), 1.62 (95 % CI 1.03, 2.55) and 1.36 (95 % CI 0.93, 1.97), respectively, with the IDF criteria. Additionally, abdominal obesity using the AHA/NHLBI criteria was significantly associated with the incidence of stroke (HR: 1.60; 95 % CI 1.01, 2.52). However, among rural elderly males, neither MetS nor its components predicted new-onset CVD.

**Conclusions:**

MetS is correlated with high incidence of CVD among rural elderly female, and only using the NCEP ATP III criteria to define MetS could make the incidence of CVD obvious difference. In order to reduce rural elderly CVD, effective measures to prevent, diagnose, and treat MetS should be enacted in a timely manner, especially among females.

## Background

With the advent of an aging population, elderly suffered high rates of Noncommunicable diseases (NCDs), which included cardiometabolic diseases such as cardiovascular diseases (CVDs), stroke and diabetes, and their major risk factors. Growing evidence confirm that CVDs increase cardiovascular mortality and all-cause mortality, especially among older subjects [1]. However, in a few years’ time, high-income countries had relatively decreased CVD mortality rate whereas the rate increased in low- and middle-income countries. There are various reasons that may result in this discrepancy and possible relate to environmental, social, and commercial determinants of health [2]. Due to the improvement of preventing, cardiovascular risk factors were effective diagnosed and treated, therefore, resulting in the falling of CVD mortality in developed countries [3, 4]. However, recently, as metabolic disorders such as obesity, elevated blood pressure, abnormal blood glucose, and dyslipidaemia became prevalent in rural China. Likewise, it accompany with an elevating CVD mortality among rural Chinese elderly [5–7]. Metabolic syndrome (MetS) is a group of cardiometabolic risk factors and comorbidities conveying high risk of both CVD and cerebrovascular disease [8]. MetS and CVD are contributing to large socioeconomic costs with their resulting morbidity and mortality around the world.

The possible relationship between MetS and the risk of CVD in different populations was still controversial among elderly.

Some reported that there was no significant relationship between MetS and CVD in the elderly whereas others insisted that MetS significantly increased the risk of fatal and non-fatal CVD [9, 10]. Additionally, Dekker JM and colleagues reported a gender discrepancy in the association between MetS and CVD [11]. There is an obvious paucity of studies estimating the predictive effect of MetS and its components on CVD in rural elderly populations. Most of the existing studies are limited by their small sample sizes and cross-sectional design. As far as we know, no population cohort study has investigated the prospective relationship between MetS and CVD in an elderly population from rural areas.

We intended to estimate whether MetS and its components at baseline can be an effective predictor of CVD among elderly subjects from rural China and whether or not there is gender discrepancy in the association between MetS and CVD. Additionally, due to the variation of MetS criteria, we compared three commonly used criteria (i.e., the updated International Diabetes Federation [IDF]’s criteria, the NCEP ATP III criteria and the Blood Institute (AHA/NHLBI) criteria) to determine which one was better at predicting CVD in rural elderly subjects.

## Methods

### Data source and study subjects

The Northeast China Rural Cardiovascular Health Study (NCRCHS) was a community-based prospective cohort study. The survey was performed in Liaoning province in China from 2012 to 2013, and follow-up occurred from 2015 to 2017. The rationale, design and methods were described in detail previously [7, 12]. The whole study included questionnaire completion and physical examination. To control for potential sources of bias, we used a standardized questionnaire which was listed previously [7, 12]. The Ethics Committee of China Medical University approved this study (Shenyang, China AF-SDP-07-1, 0–01). A written informed consent was signed by all participants. The total follow-up rate was 86.6 % and the median follow-up years was 4.66. In this study, only subjects ≥ 60 years were enrolled (n = 2486).

### Definition of MetS

MetS in the present study was defined using three criteria (Table 1).

**Table 1:** The definition of Metabolic syndrome

	IDF definition	NCEP ATP III	the AHA/NHLBI
	Central obesity plus any two of the following four additional factors	Three or more of the following five factors	Three or more of the following five factors
1	Central obesity: waist circumference ≥ 90 cm in men and ≥ 80 cm in women	central obesity: waistcircumference ≥ 102 cm in men and ≥ 88 cm in women	Same as NCEP APT III
2	Hypertriglyceridemia triglyceride level ≥ 1.7mmol/L;	Same as IDF	Same as NCEP APT III
3	High blood pressure ≥ 130/85mm Hgor treatment of previously diagnosed hypertension;	Same as IDF	Same as NCEP APT III
4	Reduced high-density lipoprotein (HDL)-cholesterol < 1.03mmol/L in men and < 1.29mmol/L in women, or specific treatment for these lipid abnormalities;	Same as IDF	Same as NCEP APT III
5	Hyperglycemia: fasting glucose level of ≥ 5.6mmol/L or treatment of previously diagnosed type 2 diabetes	Hyperglycemia: fasting glucose level of ≥ 6.1mmol/L or treatment of previously diagnosed type 2 diabetes	Hyperglycemia:fasting glucose level of ≥ 5.6mmol/L or previously diagnosed type 2 diabetes.

**Table 2:** Demographic, anthropometric and plasma biochemical characteristics of subjects at baseline

Characteristics	Female (*n* = 1245)	Male (*n* = 1241)	*P* value
**Age (years)**	66.49 ± 5.50	67.12 ± 5.89	0.006
**Age**			0.016
60–70	957(76.9)	892(71.9)	
70–80	255(20.5)	306(24.7)	
≥80	33(2.7)	43(3.5)	
**Ethnicity**			0.049
Han	1182(94.9)	1196(96.4)	
Others ^a^	63(5.1)	45(3.6)	
**Education status**			< 0.001
Primary school or below	1066(85.6)	801(64.5)	
Middle school	152(12.2)	359(28.9)	
High school or above	27(2.2)	81(6.5)	
**Marriage status**			< 0.001
Married	974(78.3)	1060(85.5)	
Single or divorced	2(0.2)	26(2.1)	
Widowed	268(21.5)	154(12.4)	
**Physical activity**			< 0.001
Light	794(64.6)	617(50.2)	
Moderate	202(16.4)	203(16.5)	
Severe	233(19.0)	410(33.3)	
**Annual income (CNY/year)**			0.380
≤ 5000	296(23.8)	282(22.7)	
5000–20,000	703(56.6)	687(55.4)	
> 20,000	244(19.6)	271(21.9)	
**Current smoking status**	298(23.9)	622(50.1)	< 0.001
**Current drinking status**	56(4.5)	491(39.6)	< 0.001
**Family history of stroke**	213(17.1)	195(15.7)	0.188
**Family history of coronary heart disease**	163(13.1)	98(7.9)	< 0.001
**Family history of hypertension**	246(19.8)	200(16.1)	0.010
**Antihypertension medication in 2 weeks**	301(35.08)	219(25.26)	< 0.001
**Antidiabetic medication in 2 weeks**	77(36.67)	33(22.45)	< 0.001
**Antidyslipidemic medication in 2 weeks**	49(4.43)	26(3.07)	0.654
**BMI (kg/m**^**2**^**)**	24.53 ± 3.91	24.10 ± 3.39	0.004
**SBP (mmHg)**	151.08 ± 24.56	152.08 ± 23.24	0.296
**DBP (mmHg)**	80.62 ± 11.84	83.42 ± 11.34	< 0.001
**WC (cm)**	82.13 ± 10.32	82.91 ± 9.57	0.053
**WC (cm) (NCEP ATP III and AHA/NHLBI)**^b^	353(28.7)	33(2.7)	< 0.001
**WC (cm) (IDF)**^c^	726(59.0)	306(25.0)	< 0.001
**TC (mmol/L)**	5.70 ± 1.09	5.17 ± 1.03	< 0.001
**TG (mmol/L)**	1.77 ± 1.24	1.38 ± 1.16	< 0.001
**LDL-C(mmol/L)**	3.27 ± 0.85	2.90 ± 0.81	< 0.001
**HDL-C (mmol/L)**	1.44 ± 0.37	1.44 ± 0.45	0.898
**FPG (mmol/L)**	6.15 ± 1.97	5.97 ± 1.60	0.015
**FPG (mmol/L) (NCEP ATP III)**^d^	382(30.9)	333(27.1)	0.021
**FPG (mmol/L) (AHA/NHLBI and IDF)**^e^	671(54.2)	654 (53.2)	0.311

^a^Including some ethnic minorities in China, such as Mongol and Manchu.

^b^ Waist circumference ≥ 102 cm in men and ≥ 88 cm in women.

^c^ Waist circumference ≥ 90 cm in men and ≥ 80 cm in women.

^d^ Fasting glucose level of ≥ 6.1 mmol/L.

^e^ Fasting glucose level of ≥ 5.6 mmol/L.

*BMI* body mass index, *WC* waist circumference, *CNY* China Yuan (1CNY = 0.161 USD), *SBP* systolic blood pressure, *DBP* diastolic blood pressure, *TG* triglyceride, *LDL-C* low-density lipoprotein cholesterol, *HDL-C* high-density lipoprotein cholesterol, *FPG* fasting plasma glucose. *AHA* American Heart Association; *IDF* International Diabetes Federation; *NCEP ATP III* National Cholesterol Education Program Adult Treatment Panel III; *NHLBI* National Heart, Lung, and Blood Institute.

### Diagnosis of CVDs

CVD was defined as a composite of new onset stroke or CHD during the follow-up period. The specific incidences of stroke and CHD were also determined. The WHO Multinational Monitoring of Trends and Determinants in Cardiovascular Disease (MONICA) criteria was used to define stroke in present study [13, 14]. Rapidly developing signs of focal or global disturbance of cerebral function, lasting more than 24 h (unless interrupted by surgery or death) with no apparent non-vascular causes were used to describe stork. Haemorrhagic stroke included subarachnoid haemorrhage or intracerebral haemorrhage. Ischaemic stroke was defined as subjects with diagnosis of thrombosis or embolism. Transient ischaemic attack and chronic cerebral vascular disease were excluded. CHD was defined as a diagnosis of hospitalized angina, hospitalized myocardial infarction, CHD death or any revascularization procedure [15].

### Covariates

Trained researchers collected participants’ sociodemographic characteristics (age, gender, education and marital status), lifestyle (current smoking and drinking), history of chronic diseases and physical activity through face-to-face interviews as described in detail previously [7, 12]. Intensity of physical activity was divided in to three groups: light, moderate and severe. Educational level was divided into three groups: ≤ primary school, middle school, and ≥ high school. Annual income of the family was categorized into ≤ 5000 CNY/year, 5000–20,000 CNY/year and > 20,000 CNY/year. Family history of chronic diseases diagnosed by a physician was self-reported, including hypertension, CHD and stroke.

### Data analysis

Continuous variables were reported as the mean values and standard deviations whereas categorical variables were reported as numbers and percentages. ANOVA, t-test, non-parameter test or the χ2-test were used to calculate the difference among categories as appropriate. The associations of MetS and its components with the risk of CHD, stroke and total CVD incidence were analysed using Cox proportional hazards models and listed by hazard ratios (HRs) and 95 % confidence intervals (CIs). All statistical analyses were performed using SPSS version 17.0 software (Chicago, IL), and P values less than 0.05 were considered statistically significant.

## Results

The present study used data from 2434 subjects (aged ≥ 60 years) who received clinical examinations at baseline and follow-up. Table 2 shows the characteristics of the 2486 elderly subjects (1245 female and 1241 male). Except for annual income, family history of stroke, antidyslipidemic medication in 2 weeks, SBP, WC, and HDL-C, statistically significant differences were found between males and females in other demographic, anthropometric and clinical characteristics. As age increased from 60 to 70 years to > 80 years, the prevalence of MetS defined by the NCEP ATP III criteria and hypertriglyceridemia significantly decreased. Elevated blood pressure increased in the relatively older age group compared to 60–70 years.

**Fig. 1:**
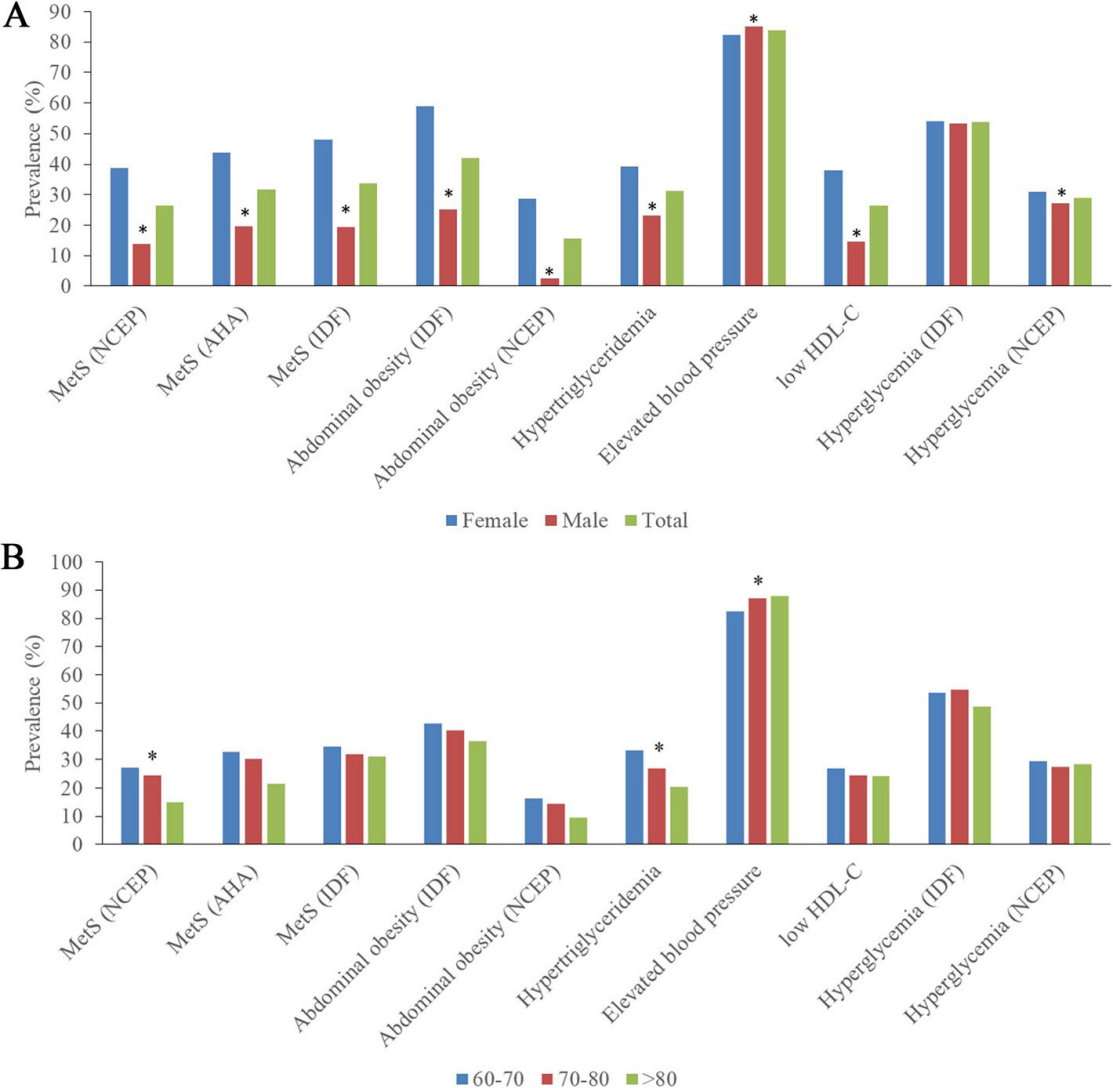
Showed the prevalence of MetS and individual metabolic components according to gender (A) and different age groups. * means *P* < 0.05 vs. female

Figure 1 shows the prevalence of MetS according to different criteria and its metabolic components in different genders and ages. Elderly females were significantly more likely to have MetS, abdominal obesity, hypertriglyceridemia and lower HDL-C levels; they were also less likely to have elevated blood pressure. There was no significant difference in hyperglycaemia between males and females (*P* = 0.347).

Tables 3 and 4 show the estimated association between different CVD conditions and MetS or components of MetS by different definitions. The data showed that only NCEP ATP III-defined MetS could predict CVD in females [HR (95 % CI): 1.45 (1.00, 2.10)] but not in males [HR (95 % CI): 1.25 (0.80, 1.97)]. Similarly, IDF-defined MetS could predict new-onset stroke in females [HR (95 % CI): 1.62 (1.03, 2.55)] but not in males [HR (95 % CI): 1.35 (0.88, 2.06)]. Regarding the metabolic components separately, abdominal obesity defined by the NCEP ATP III and AHA/NHLBI criteria was associated with stroke among females [HR (95 % CI): 1.60 (1.01, 2.52)]. There was lack of predictive effect of other metabolic components on CVD in both females and males.

**Table 3:** Hazard ratio and 95%CI of CVD for MetS and the number of components of MetS by the NCEP, AHA/NHLBI and IDF criteria in elderly female from rural China

	CHD(***n***=54)		Stroke(***n***=85)		CVD(***n***=119)	
	Crude HR (95%CI)	Adjusted HR (95%CI)	Crude HR (95%CI)	Adjusted HR (95%CI)	Crude HR (95%CI)	Adjusted HR (95%CI)
**MetS by different criteria (ref=non- MetS)**						
NCEP ATP III	1.38 (0.80,2.37)	1.27 (0.73,2.21)	1.52 (0.99,2.33)	1.54 (0.99,2.40)	**1.45** **(1.02,2.08)**	**1.45** **(1.00, 2.10)**
AHA/NHLBI	1.41 (0.82,2.44)	1.33 (0.77,2.32)	1.42 (0.93,2.18)	1.44 (0.92,2.25)	1.35 (0.94,1.93)	1.36 (0.94, 1.97)
IDF	1.09 (0.64,1.88)	1.10 (0.89,1.36)	**1.56** **(1.01,2.41)**	**1.62** **(1.03,2.55)**	1.36 (0.95,1.95)	1.36 (0.93, 1.97)
**Metabolic components (ref= no)**						
Elevated Blood pressure	2.06 (0.82,5.21)	1.69 (0.66,4.33)	1.43 (0.76,2.70)	1.29 (0.67,2.45)	1.49 (0.87,2.56)	1.31 (0.76,2.28)
Hypertriglycerides	1.12 (0.65,1.93)	1.12 (0.65,1.94)	1.03 (0.67,1.60)	1.05 (0.67,1.64)	1.20 (0.84,1.73)	1.22 (0.84,1.76)
Low HDL-C	1.38 (0.81,2.37)	1.38 (0.80,2.39)	1.21 (0.79,1.86)	1.30 (0.83,2.03)	1.31 (0.91,1.87)	1.42 (0.98,2.05)
Abdominal obesity (NCEP ATP III and AHA/NHLBI) ^a^	1.04 (0.58,1.87)	0.95 (0.52,1.72)	**1.55** **(1.00,2.41)**	**1.60** **(1.01,2.52)**	1.20 (0.82,1.76)	1.18 (0.80,1.75)
Abdominal obesity (IDF) ^b^	1.05 (0.60,1.84)	0.97 (0.55,1.71)	1.46 (0.93,2.31)	1.41 (0.88,2.25)	1.25 (00.86,1.82)	1.18 (0.80,1.73)
High FPG (NCEP ATP III) ^c^	0.76 (0.41,1.40)	0.75 (0.40,1.39)	1.34 (0.86,2.08)	1.32 (0.83,2.11)	1.19 (0.81,1.73)	1.18 (0.80,1.75)
High FPG (AHA/NHLBI and IDF) ^d^	0.94 (0.55,1.61)	0.99 (0.57,1.72)	1.33 (0.85,2.06)	1.36 (0.86,2.16)	1.25 (0.86,1.80)	1.30 (0.89,1.90)

Adjusted for sex, age, marital status, education, physical activity, currently smoking (yes, no), currently drinking (yes, no), family history of CHD, family history of stroke and family history of hypertension.

^a^ Waist circumference ≥102 cm in men and ≥88 cm in women.

^b^ Waist circumference ≥90 cm in men and ≥80 cm in women.

^c^ Fasting glucose level of ≥6.1 mmol/L.

^d^ Fasting glucose level of ≥5.6 mmol/L.

**Table 4:** Hazard ratio and 95 %CI of CVD for MetS and the number of components of MetS by the NCEP, AHA/NHLBI and IDF criteria in elderly male from rural China

	CHD(*n* = 39)		Stroke(*n* = 122)		CVD(*n* = 149)	
	**Crude HR (95 %CI)**	**Adjusted HR (95 %CI)**	**Crude HR (95 %CI)**	**Adjusted HR (95 %CI)**	**Crude HR (95 %CI)**	**Adjusted HR (95 %CI)**
**MetS by different criteria (ref = non- MetS)**						
NCEP ATP III	1.36(0.60,3.08)	1.29(0.55,2.99)	1.19(0.74,1.92)	1.28(0.79,2.09)	1.16(0.74,1.80)	1.25(0.80,1.97)
AHA/NHLBI	1.21(0.57,2.55)	1.14(0.53,2.44)	1.31(0.87,1.99)	1.41(0.92,2.15)	1.23(0.84,1.81)	1.32(0.90,1.96)
IDF	1.10(0.51,2.41)	0.98(0.44,2.18)	1.43(0.95,2.16)	1.35(0.88,2.06)	1.36(0.94,1.98)	1.28(0.87,1.89)
**Metabolic components (ref = no)**						
Elevated Blood pressure	0.60(0.27,1.30)	0.53(0.24,1.19)	0.99(0.60,1.63)	0.98(0.59,1.63)	0.88(0.57,1.36)	0.86(0.55,1.35)
Hypertriglycerides	1.15(0.56,2.38)	1.24(0.59,2.58)	1.25(0.84,1.87)	1.33(0.89,2.00)	1.13(0.78,1.64)	1.24(0.85,1.81)
Low HDL-C	1.58(0.72,3.44)	1.19(0.54,2.64)	0.89(0.52,1.50)	0.90(0.52,1.53)	0.99(0.62,1.57)	0.97(0.61,1.55)
Abdominal obesity (NCEP ATP III and AHA/NHLBI) ^a^	2.07(0.50,8.62)	2.17(0.52,9.14)	0.56(0.14,2.27)	0.56(0.14,2.27)	1.06(0.39,2.85)	1.10(0.41,2.98)
Abdominal obesity (IDF) ^b^	1.21(0.60,2.45)	1.12(0.54,2.31)	1.17(0.79,1.74)	1.08(0.71,1.643	1.20(0.84,1.72)	1.12(0.77, 1.62)
High FPG (NCEP ATP III) ^c^	1.70(0.89,3.26)	1.72(0.88,3.34)	1.10(0.75,1.63)	1.15(0.77,170)	1.22(0.86,1.72)	1.26(0.89, 1.79)
High FPG (AHA/NHLBI and IDF) ^d^	1.15(0.60,2.19)	1.14(0.59,2.19)	1.16(0.81,1.67)	1.18(0.82,1.71)	1.19(0.86,1.65)	1.22(0.88,1.71)

Adjusted for sex, age, marital status, education, physical activity, currently smoking (yes, no), currently drinking (yes, no), family history of CHD, family history of stroke and family history of hypertension. ^a^ Waist circumference ≥ 102 cm in men and ≥ 88 cm in women. ^b^ Waist circumference ≥ 90 cm in men and ≥ 80 cm in women. ^c^ Fasting glucose level of ≥ 6.1 mmol/L. ^d^ Fasting glucose level of ≥ 5.6 mmol/L.

## Discussion

In the present study, NCEP ATP III-defined MetS at baseline was associated with a significantly higher risk of new-onset CVD among elderly females. Similarly, IDF-defined MetS was correlated with new-onset stroke among elderly females from rural China. Regarding individual metabolic components, only abdominal obesity defined by the NCEP ATP III criteria was correlated with new-onset stroke.

During the past decades, with the rapid development of economic growth, changes in lifestyle and longer life expectancy, the geriatric population has increased worldwide. Together with the ageing population, age-related metabolic disorders, such as elevated blood press, hyperglycaemia, obesity and dyslipidaemia, have gradually become even prevalent. The high rates of cardiometabolic risk factors caused dramatically increased cardiovascular and cerebrovascular problems which resulted in a higher morbidity and mortality in elderly subjects. The prevalence of MetS in the elderly population varies from 11 to 43 % (median 21 %) and 23–55 % (median 31 %) according to the NCEP ATP III criteria [16]. Obesity and hypertension are the most prevalent individual components. Due to effective propagation, prevention and treatment, cardiometabolic disorders showed a downward trend in developed countries during the past decades. However, these disorders still occur frequently in rural or developing areas. Our previous data showed that during 2012–2013, the prevalence of hypertension (74.8 %), diabetes (14.9 %), dyslipidaemia (67.4 %), obesity (39.9 %), and stroke (18.9 %) among the elderly was significantly high in rural China [17]. In the present study, the prevalence of MetS defined by the IDF criteria was 35.5 and 28.4 % defined by the NCEP ATP III criteria; this was similar to the data from urban cities such as Beijing [MetS by the NCEP criteria was 30.5 %; MetS by the IDF criteria was 46.3 %] [18]. Due to the high prevalence of MetS among rural elderly subjects, it is necessary to estimate the possible effect of MetS on new-onset CHD, stroke, and CVD to better prevent and control cardiovascular mortality and morbidity.

Numerous previous studies have estimated the relationship between MetS and CHD or stroke, with inconsistent results. One cross-sectional study also conducted in rural China concluded that NCEP-ATP III-defined MetS was more suitable than IDF and Chinese Diabetes Society criteria for screening and estimating the risk of CHD and stroke from MetS, especially in men [19]. Similarly, elderly subjects from Beijing with MetS had significantly higher risk of CHD, stroke, PAD and CVD [18]. However, in the Prospective study of Pravastatin in the Elderly at Risk (PROSPER) and British Regional Heart Study (BRHS) studies, weak or no association between MetS and vascular risk in elderly subjects was found using the NCEP ATP III criteria [20]. Elderly subjects usually had a variety of cardiovascular risks or higher rate of established CVD. Therefore, when we intended to evaluate the association between MetS and the risk of developing CVD, we should consider the possible impact of cardiovascular risks and CVD at baseline. Ana Teresa Timóteo and colleagues reported that among subjects (a mean age of 65 ± 9 year) with a high cardiovascular risk, the presence of MetS at baseline was not associated with cerebral or cardiac events in long-term follow-up [21]. Most of the previous studies estimated that the relationship between MetS and CVD were cross-sectional analyses that restricted their accuracy. As a prospective study, we found that MetS defined by the NCEP ATP III and IDF criteria at baseline was correlated with new-onset CVD and stroke, respectively, in women but not men, which helped to confirm the effect of MetS on CHD, stroke or CVD. A previous study also claimed that the association between CVD and MetS defined by the NCEP ATP III and AHA/NHLBI criteria was more pronounced compared with MetS defined by the IDF criteria [22]. However, a study performed in China concluded that IDF-defined MetS was more strongly associated with CHD than the NCEP- or revised NCEP-defined MetS but weakly or not associated with stroke, which was inconsistent with our results [23]. In our study, abdominal obesity at baseline increased new onset stroke in elderly females but not males. This finding was consistent with many previous studies. Data from the National Stroke Screening Survey in 2012 and the 2010 Chinese population from the sixth National Census of Populations showed that compared to elderly males, elderly females with stroke were more likely to have obesity, diabetes, elevated LDL-C and atrial fibrillation [24]. This finding may partially be due to the significantly lower rate of abdominal obesity in males compared to females (2.9 % vs. 29.4 %, P < 0.001) in the present study, which led to the predictive effect of MetS on new-onset stroke being insignificant.

One interesting finding in the present study was that the predictive effect of MetS on new-onset CHD, stroke or CVD was significant in elderly females but not elderly males. Previously, one meta-analysis that enrolled 21 studies claimed that females with MetS had significantly higher relative risk of CVD compared with males (RR: 2.10 vs. 1.57) [25]. First, this finding might be due to the gender discrepancy in the prevalence of MetS in elderly subjects. Trevisan et al. reported that of subjects aged ≥ 50 years, women had a significantly higher prevalence of MetS than men [26]. Similarly, a study in the USA of elderly subjects aged ≥ 70 years showed that MetS (NCEP ATP III) was more prevalent among women than men [27]. Data inferred that among elderly subjects, the prevalence of MetS was relatively higher among females compared with males, whereas in general epidemiological studies, males had a significantly higher rate of MetS than females [28]. Women had a significantly higher rate of MetS at baseline, which might result in a significant predictive effect of MetS on new-onset CVD in the future. Second, diabetes is a stronger risk factor in women than in men, increasing the risk of CHD by three- to sevenfold in diabetic females compared with a two- to threefold increase in risk for diabetic males [29]. Insulin resistance is a hallmark of MetS and manifests with impaired fasting glucose. In the Framingham study, females with impaired fasting glucose had increased CHD risk to a similar degree as established diabetes whereas this association did not exist among males [30]. In addition, there was a higher risk of CVD associated with MetS in studies that included participants with diabetes compared with those that excluded participants with diabetes [31–33]. Furthermore, in subgroup analyses of five studies that were restricted to participants with diabetes, data inferred an increased risk of CVD associated with MetS [25]. These results call into question whether the individual components convey the same risk as the syndrome as a whole. It is possible that some specific components, such as hyperglycaemia or increasing numbers of metabolic disorders, might affect the predictive effect of MetS on CVD. In our study, we found that elderly females were more likely to have abdominal obesity, hypertriglyceridemia, low HDL-C, and hyperglycaemia than elderly males. However, the HDL-C level did not show significant difference between elderly females and males. One possible explanation might be due the menopause status which plays an important role in changing lipid profile. Compared with premenopausal women, postmenopausal women had a more atherogenic lipid profile with lower HDL-C [34]. Tian Li and colleagues also found that, with the increase of age, the difference of gender discrepancy in HLD-C decreased dramatically among Chinese subjects. The level of HDL-C in postmenopausal women with normal blood lipids are similar to those in men with normal lipids [35]. Furthermore, Kyung-Hyun Cho and colleagues claimed that income level had close relationship with HDL-C level. They figured out that in both female and male, a significant increase in the average income was associated with a concomitant increase in HDL-C level. A lower income level was associated directly with a lower HDL-C level, which suggested that poverty was associated with a lower HDL-C [36]. Nevertheless, the increased metabolic abnormalities might result in the discrepancy of predictive efficiency of MetS as a whole on CVD. Third, except for baseline MetS, there are many possible confounders that have been analysed in our model that may cause the association between MetS and CVD to be insignificant. In the present study, after adjusting for possible confounders, MetS (OR: 1.33), female gender (OR: 1.42), increasing age (OR: 1.04), and family history of hypertension (OR: 1.54) were associated with increased risk of new-onset CVD. We further analysed the characteristics of female and male participants separately and found that elderly females had a significantly higher rate of family history of hypertension than elderly males (19.7 % vs. 16.2 %, P = 0.012). Therefore, the association between MetS and CVD may be more pronounced in elderly females. Finally, there is another possible explanation regarding the gender discrepancy that may be relevant to hormonal influences. Premenopausal women have a significantly lower absolute risk of death from CHD within a limited observational period whereas menopause escalates CHD risk threefold [37, 38]. In our present study, we enrolled elderly females aged ≥ 60 years who were in postmenopausal status. Oestrogen is a protective factor, and menopause is associated with a significant decline in circulating oestrogen levels. However, the Women’s Health Initiative (WHI) and the Heart and Estrogen/Progestin Replacement Study (HERS) showed that menopausal hormone therapy failed to prevent CVD in women. Therefore, further research is required to confirm whether hormones can account for the gender discrepancy of the predictive effect of MetS on CVD.

MetS is significantly associated with high incidence of CVD among rural elderly. In order to better control MetS among rural Chinese elderly, we should emphasize the harm of dyslipidaemia and the importance of lipid-lowering treatment. Among patients with established cardiovascular disease, type 2 diabetes, obesity or metabolic syndrome, atherogenic dyslipidaemia (AD) like hypertriglycerides and low HDL-C is common and is correlated with macrovascular and microvascular residual risk [39]. First, lifestyle optimization should be recommended to the rural elderly, like proper physical activity, smoke cessation, and lessen salt intake. Additionally, pharmacotherapy is often required like LDL-lowering therapy with statins (± ezetimibe) should be recommended [40].

## Limitation

There are some limitations in the present study. First, some participants were lost to follow-up, which might cause bias in the predictive effect of MetS on CHD, stroke or CVD. Second, HDL-C, LDL-C, triglyceride, and fasting plasma glucose were measured only once, which might be imprecise and result in random errors. Third, in the present study, we did not use the WHO definition or that of the European Group for the Study of Insulin Resistance due to a lack of data on insulin resistance. Finally, the detail message like variety and dosage of antihypertensive, antidiabetic and antidyslipidemic medication was not collected in the questionnaire which might be closely related to the gender discrepancy.

## Conclusions

In summary, our findings suggest that MetS is significantly associated with CVD and stroke when using the NCEP ATP III and IDF criteria to define MetS, respectively. Among a rural elderly female Chinese population, abdominal obesity defined by the NCEP ATP III criteria was more prevalent than other components of MetS for HRs of stroke. The NCEP ATP III criteria may be more suitable for estimating the predictive effect of MetS on CVD, whereas the IDF criteria are more suitable for predicting stroke among rural elderly females in China.

## Data Availability

Enquiries regarding the availability of primary data should be directed to the principal investigator Professor Yingxian Sun (sunyingxiancmu1h@163.com).

## Abbreviations

CVD: Cardiovascular disease
CHD: Coronary heart disease
MetS: Metabolic syndrome
BMI: Body mass index
SBP: Systolic blood pressure
DBP: Diastolic blood pressure
FPG: Fasting plasma glucose
TC: Total cholesterol
LDL-C: Low-density lipoprotein cholesterol
HDL-C: High-density lipoprotein cholesterol
TG: Triglyceride
